# A pilot program of knowledge translation and implementation for newborn resuscitation using US Peace Corps Volunteers in rural Madagascar

**DOI:** 10.1186/s12992-016-0207-3

**Published:** 2016-11-16

**Authors:** Kristin Close, Michele Karel, Michelle White

**Affiliations:** 1 Department of Medical Capacity Building, Mercy Ships, Port of Toamasina, Toamasina, Madagascar; 2 US Peace Corps Volunteer, Antananarivo, Madagascar

**Keywords:** Global Health, Newborn resuscitation, Education, Medical missions, Perinatal outcome

## Abstract

**Background:**

Prevention of adverse perinatal outcome using the Helping Babies Breathe (HBB) neonatal resuscitation algorithm can reduce perinatal mortality in low income settings. Mercy Ships is a non-governmental organisation providing free healthcare education in sub-Saharan Africa and in an attempt to reach more rural areas of Madagascar with our neonatal resuscitation training we designed a novel approach in collaboration with US Peace Corps Volunteers (PCV). PCVs work in rural areas and contribute to locally determined public health initiatives.

**Method:**

We used a model of knowledge translation and implementation to train non-medical PCVs in HBB who would then train rural healthcare workers. Bulb suction and a self-inflating bag were donated to each health centre. We evaluated knowledge translation and behaviour change at 4 months using the Kirkpatrick model of evaluation.

**Results:**

Ten PCVs received training and then trained 42 healthcare workers in 10 rural health centres serving a combined population of over 1 million. Both PCVs and rural healthcare workers showed significant increases in knowledge and skills (*p* < 0.001). The commonest behaviour changes persisting at 4 months were adequate preparation before delivery; use of rubbing and drying as a means of stimulation instead of foot tapping or back slapping; and use of the self-inflating bag to give respirations. Anecdotal evidence of changes in neonatal outcome were reported in several health care centres.

**Conclusion:**

Our study demonstrates that non-medically trained PCVs can be used to successfully train rural healthcare workers in newborn resuscitation using the HBB algorithm and this results in improvements in personal and organizational practice at 4 months, including anecdotal evidence of improved patient outcome. Our novel method of training, including the provision of essential equipment, may be another tool in the armamentarium of those seeking to disseminate good practice to the most rural areas.

**Electronic supplementary material:**

The online version of this article (doi:10.1186/s12992-016-0207-3) contains supplementary material, which is available to authorized users.

## Background

Every year approximately 136 million babies are born globally and an estimated 10% of these newborns have absent or poor respiratory effort requiring assistance to achieve cardiorespiratory stability [1, 2]. Most only require simple assistance, with less than 1% requiring advanced resuscitation such as intubation, chest compressions and medications [1]. However these estimates are based on only a few reports [1–5] none of which reflect sub-Saharan Africa where the burden of perinatal deaths and morbidity is considered to be the highest in the world [6, 7]. In Madagascar the neonatal mortality is 22 per 1000 live births; less than 35% of births occur at a healthcare facility; and less than half of all births are attended by a skilled birth attendant [8].

Strategies for prevention of adverse perinatal outcome can be divided in to 3 phases: (i) primary prevention of insult; (ii) secondary prevention after the insult; (iii) tertiary prevention of complications [9]. While primary prevention is likely to have the biggest impact on outcome, secondary prevention using the Helping Babies Breathe (HBB) neonatal resuscitation algorithm [10], and other simple resuscitation techniques can effectively reduce perinatal mortality [11–14] even in resource limited settings. HBB uses a low-tech, neonatal simulator (NeoNatalie, Laerdal Medical) to train birth attendants (of various skill levels), and is included in the WHO Essential Newborn Care Course [15].

Evaluation of new avenues for learning and collaboration are needed if widespread knowledge translation, especially in rural areas, is to be achieved. Current methods either require rural healthcare workers to travel from their facility to attend training elsewhere therefore leaving the facility without trained personnel; or require trained persons from the city to spend time travelling to the rural areas, which is time consuming and also takes the trainers away from front-line work. The US Peace Corps has volunteers working in remote rural health centres to support public health initiatives determined by the collaborative efforts of the PCV and the community in which they work. PCVs are not medically trained yet well accepted and integrated into the rural health setting, culture, and language, making them ideally placed to lead a knowledge translation program. Since the HBB algorithm contains simple techniques, able to be performed by non-medically trained persons, we hypothesised that Mercy Ships could train PCVs, who could, in turn, successfully train health centre workers. Mercy Ships is a non-governmental organisation providing free surgeries and medical education in port cities of sub-Saharan Africa.

If successful, this approach could help deliver training more easily and with wider reach across rural communities.

## Methods

The study was approved by the Mercy Ships Institutional Review Board, number MS2016002.

Using a model of knowledge translation, implementation and on going monitoring we aimed to train 10 PCVs in the HBB algorithm, and then the 10 PCVs, in turn, would train rural healthcare workers. We aimed to assess the educational efficiency and knowledge translation at 4 months using the Kirkpatrick model of evaluation.

In collaboration with the US Peace Corps, 10 rural health centres (Centre de Santé de Base) were identified, in 3 different regions of Madagascar. Sites were identified pragmatically based on ability to access the site within 2 days travel (by plane and/or car) of the port city of Toamasina where the Mercy Ships hospital ship was based. Each PCV was required to have been working at the rural health centre for at least 6 months so that a relationship of trust within the community and other healthcare workers was already established.

Ten PCVs attended a 1 day of HBB training on board the *Africa Mercy* Hospital Ship in January 2015. Two Mercy Ships staff (one an approved HBB trainer, the other a registered nurse and Neonatal Resuscitation Program instructor for the American Academy of Pediatrics) with at least 2 years experience of teaching and training in Africa conducted the HBB training. After a focus group discussion on challenges of implementation, each PCV was given a NeoNatalie simulator, re-usable manual self-inflating resuscitation bag, and bulb suction. PCVs returned to their health centres and were asked to train 4–6 health care workers each during the next month. Official HBB training materials including knowledge and skills assessments [16] were provided in French and English and translated locally into Malagasy for use in the rural setting.

Basic data on the functionality (workforce, equipment and infrastructure) of the health centres was collected.

We used the New World Kirkpatrick model of evaluation of training to assess knowledge translation and implementation as shown in Table 1 [17].

**Table 1:** Kirkpatrick model for evaluating effects of educational courses

Level 1: Reaction	Participants perception of the course (enjoyment, relevance and engagement)
Level 2: Learning	Acquired knowledge, skills, attitude, confidence, commitment
Level 3: Behaviour	Translation of knowledge and skills into routine personal practice
Level 4: Results	The ultimate goal; improved patient outcome

### Level 1 (reaction)

The program was assessed using a Mercy Ships standard participant program evaluation form. The form asks participants to rate their enjoyment of the course; if the course has improved their knowledge; if the course has improved their ability to perform their work; whether they would recommend the program to others. The form also asks them to list what they found difficult about the course and suggestions for improvement. This was completed by PCVs on the day of training and by healthcare workers after they had been trained by the PCVs.

### Level 2 (learning)

In order to measure whether the program resulted in changes in knowledge and skills, the PCVs pre and post-course knowledge was assessed with the HBB 17 item theory test (choose best one response out of four) [16] administered at the beginning and the end of the 1 day training. PCVs subsequently administered the HBB theory test and 18 point practical skills test [16] to the healthcare workers before and after their training.

### Level 3 (behaviour) and Level 4 (patient outcome)

To measure the effective transfer and dissemination of the information by PCVs to the rural healthcare workers we used quantitative and qualitative methodology.

Quantitative: The level 1 reaction and level 2 learning of the healthcare workers as described above formed the quantitative analysis of the ability of the PCV to effectively transfer knowledge and skills to rural healthcare workers.

Qualitative: At 4 months we conducted observational site-visits and used questionnaires, structured interviews and discussion groups to assess level 3 changes in behaviour; evaluate level 4 improvements in patient outcome; and to identify facilitators and inhibitors to change. Because of resource constraints, interviews were not tape recorded but responses were handwritten contemporaneously. These were then manually themed by the authors. PCV interviews were conducted in English and healthcare worker interviews in Malagasy using a translator independent of the project.

Once all evaluations were completed, the Neonatalie simulators were returned to Mercy Ships to be used for further training but the bulb suction and manual self-inflating resuscitation bags (both re-usable design) were left as an equipment donation to each health centre so they were equipped to continue using the skills learnt.

Statistical analysis for quantitative data was performed using a 2-sided paired *t*-test.

## Results

Ten PCVs received training and subsequently trained 42 healthcare workers in 10 rural health centres serving a combined population of over 1 million. The 42 healthcare workers comprised 3 doctors, 5 nurses, 11 midwives, 13 community workers, 4 dispensaries (trained aides who work in the clinic pharmacy), and 6 other community members, all of whom may be required to assist at deliveries.

Basic situational analysis of the 10 health centres showed 8/10 had one doctor and one trained midwife available; 5/10 had a self-inflating bag for assisting ventilation; and only 3/10 had bulb suction. Running water and electricity was only available in 2/10 health centres but the supply was unreliable. None had oxygen available.

All 10 PCVs were available for interview and 29/42 (69%) of healthcare workers. 13 healthcare workers across the 10 sites were unavailable on the day of the follow up visits for reasons such as ‘not working that day because they worked the night shift’ or ‘visiting family in another town’.

### Level 1 reaction and Level 2 learning

For PCVs and the healthcare workers they trained, the average results for Level 1 reaction and Level 2 learning are shown in Table 2. Results were obtained for all 10 PCVs and 29/42 (69%) healthcare workers. Each group showed a statistically significant improvement in knowledge and skills after training. Some PCVs continued to assess the skill level of the healthcare workers each month when they repeated the training.

**Table 2:** Level 1 and Level 2 reactions

	Peace Corps Volunteers (*n* = 10)	Healthcare workers (*n* = 29)
Level 1 Reaction^a^ *Instructions*: *On a scale of 0*–*10*, *with one being not at all and 10 being extremely*, *please give your feedback below*.		
Was the program helpful?	9.5	8.6
Have you improved your knowledge?	9.0	8.4
Do you think the training will improve the practice at your health centre?	8.6	9.3
Would you recommend this course to others?	9.6	8.7
Will you share this information with others at your health centre	9.8	9.0
Level 2 Learning^b^		
Theory test-average pre course result (%)	75.9	60.2
Theory test-average post course result (%)	97.6	78.5
*P* value	<0.001	<0.001
Skills test-average pre course result (%)		38.5
Skills test-average post course result (%)		65.5
*P* value		<0.001

^a^Results for level 1 reaction are given as mean score from 0 to 10

^b^Results for level 2 learning are given as mean % score

### Level 3 Behaviour-translation of knowledge and skills into changes in personal practice

Site visits were conducted at 4 months after the initial PCV training. The bulb suction and self-inflating mask were identified in all sites in a suitable location either in or adjacent to the delivery room. In one site a Malagasy translation of the HBB algorithm was on display on the wall, and in five others the French algorithm was on display. All 10 PCVs were interviewed and 29 health centre workers.

The three most common responses to the question, ‘As a result of the training have you seen or initiated any change in your personal practice or your health centre?’ were, in order of frequency:

1. Use of the self-inflating bag to give respirations
2. Rubbing and drying the baby to give stimulation instead of foot tapping or back slapping
3. Preparation before delivery: of the room and equipment and having a helper available

Further comments illustrating change in personal practice (Kirkpatrick level 3) are shown in Table 3.

**Table 3:** Further examples of behaviour change (Kirkpatrick Level 3 evaluation)

• *I used to think that if the baby was born lifeless they were dead. But now I know that that is not always the case*
• *Before I didn*’*t know how to give breaths to the baby*
• *We used to use a straw to give mouth*-*to*-*mouth breaths to the baby but now we can use the* [*self*-*inflating resuscitation*] *bag*
• *I can remember being taught how to use a* [*self*-*inflating resuscitation*] *bag in my training but I had forgotten how to do it because we didn*’*t have one in our health centre*
• *We used to hit the babies on the back to help them breathe but now I know how to do it properly*
• *We used to bend the babies legs up to their chest and then push on them to make the baby breathe*
• *Now we delay cutting the cord so that the baby can benefit from getting more blood*.

### Level 4 Changes in patient outcome

Changes in patient outcome were assessed during individual structured interview and in discussion groups. In response to asking ‘do you think the training made any difference to patient outcome?’ here are three of the responses:

1. Midwife: *We* (*me and the doctor*) *were at a delivery where the baby was born blue and not breathing. The doctor is a bit disorganised and couldn*’*t remember where he had put the self*-*inflating bag but he knew where the PCV kept a spare one for training in a building next door. So he* (*the doctor*) *ran to the building*, *collected the self*-*inflating bag*, *came back and successfully resuscitated the baby. Now we always keep the bulb suction and self*-*inflating bag on the side in the delivery room*.
2. Midwife: *I was at a delivery where the baby was born blue and limp. The mother was scared because the baby had not begun to cry. I comforted the mother and said*, ‘*Have courage*, *your baby will be ok*’. *I said that because I knew what to do and I used the HBB response plan and the new equipment. They really didn*’*t respond to the stimulation but after I gave a few breaths to the baby they became pink and started crying*’
3. Nurse: *This training was so beneficial because before I didn*’*t know what to do for a baby*…….… *I would send patients to the bigger hospital but the patients couldn*’*t afford it. Now I am confident in resuscitating babies and people can trust me*.’

### Identification of inhibitors to change

In general the training was well received by all the heath care workers however 4 out of 10 PCVs reported some resistance to the teaching by older midwives in their health centres. On two occasions this was overcome when the midwives witnessed someone else using the technique to successfully resuscitate a baby who they thought could not be helped. Eight out of 10 health centres had a doctor and in contrast to the resistance among certain older midwives, all the doctors welcomed the training. Several PCVs had indicated after their initial training with Mercy Ships that they might have difficulty in persuading the older midwives to accept the new HBB method and so attempted to overcome this by holding a separate training session for those midwives and then asking the midwives to help them train the other less-educated healthcare workers. They reported they thought this was helpful and those who did not hold a separate session said they would do so if they were introducing another similar new concept in the future.

## Discussion

Our study demonstrates that non-medical Peace Corps Volunteers can be used to successfully train rural healthcare workers in newborn resuscitation using the HBB algorithm and this results in improvements in personal and organizational practice and anecdotal evidence of improved patient outcome.

Strategies for prevention of adverse perinatal outcome can be divided into 3 phases (i) primary prevention of insult with adequate fetal monitoring, correct use of the partogram, and timely obstetric intervention; (ii) secondary prevention after the insult by immediate basic resuscitation of the non-breathing baby; (iii) tertiary prevention of complications in the baby by adequate postnatal treatment [9]. Primary prevention is likely to have the biggest impact on outcome but is probably the most complex, time-consuming and expensive to address. Therefore it is important to identify simpler strategies. HBB and other simple resuscitation techniques are simple secondary prevention strategies which reduces deaths [11–14] in resource-limited settings. HBB emphasizes skilled attendants at birth, assessment of every baby, drying and swaddling to assist temperature support; stimulation to breathe and positioning of the airway; and assisted ventilation as needed, all within the ‘Golden Minute’ after birth. Our results show that HBB is simple enough to not only be taught to non-trained birth attendants (PCVs) but can also be effectively transmitted by them to others creating an efficient method of knowledge sharing and implementation. The HBB teaching focuses first on the importance of appropriate stimulation by rubbing and drying and airway positioning, with use of the self-inflating bag and mask only if these steps are ineffective. Our results indicating use of the bag and mask as the top behaviour change, may reflect the fact that the equipment given was new which may be more highly remembered or mentioned first, in contrast to the change associate with ‘stimulation’. The change associated in stimulation was one of technique rather than the use of simulation per se. None-the-less, appropriate stimulation by rubbing and drying instead of foot tapping or back slapping was the second most highly reported, and should continue to be emphasised.

The Utstein formula of survival states that patient outcome is a product of medical science, educational efficiency and implementation [9, 18]. It is estimated that all three factors in the formula contribute equally to patient outcome. HBB makes use of the best available medical science, but knowing how best to educate healthcare workers implement this knowledge is more difficult. Using Kirkpatrick Level 1 and 2 evaluations, our results demonstrate that non-medical personnel (PCVs) can effectively transfer the HBB knowledge and skills to other medical and non-medical personnel in remote rural Madagascar. Level 3 and 4 evaluations showed the bulb suction and self-inflating bags were all being used; evidence of improved practice (preparation and verification of materials before delivery; correct stimulation techniques and use of the self-inflating bag) and improved patient outcomes. We suggest that using PCVs could be used as a model for knowledge transfer in other rural settings for newborn resuscitation using the HBB algorithm. For newborn resuscitation in a resource-limited setting, a single practitioner can make the difference between life and death. In places that lack running water, electricity and oxygen, knowledge of a simple algorithm such as HBB is essential for the practitioner to save lives whether they are an untrained birth attendant, a midwife or a doctor.

Lack of equipment is commonly cited as a reason for not being able to apply knowledge in practice. Before our study only 3/10 healthcare centres had bulb suction and only 5/10 had a self-inflating bag for assisted respiration. After training, each facility was not only trained but also adequately equipped to correctly perform newborn resuscitation. Mercy Ships supplied HBB self-inflating bags, masks and suction devices that are designed to be reused. The use of the self-inflating bag and correct method of stimulation were the most commonly cited changes in practice, and this is encouraging as the concept of the ‘golden minute’ is based on performing these two actions in an appropriate and timely manner. Of the 13.6 million newborns delivered per year with absent or poor respiratory effort, nearly all will respond to simple resuscitation efforts as described by HBB. Less than 1% require advanced resuscitation with intubation, chest compressions and medications [1]. Therefore the HBB program has the potential to save millions of lives if it can be disseminated to the point of need.

Our study trained 10 PCVs and 42 healthcare workers working in rural health centres serving a population of over 1 million people, but that is likely to be only a fraction of the total numbers of traditional birth attendants in those places. The exact numbers of traditional birth attendants are unknown but are likely to be very high in countries like Madagascar where over 65% of births occur at home [19]. The role of and training for traditional birth attendants in rural settings is controversial and is beyond the scope of this study. In collaboration with the Ministry of Health and U.S. Peace Corps, we agreed to only train health centre workers and ensure they were adequately equipped to deliver care.

One of the inhibitors to change in our study was resistance among older midwives.to the ‘new HBB method’. This could have been because they were being taught by much younger and untrained PCVs. However, when they saw a baby successfully resuscitated with the HBB methods they changed their minds. Malagasy learning culture is oral and experience-based and therefore seeing something ‘work’ is an important aspect of inducing change. Relationship is another important cultural value in most African cultures, and people trust those they know, or have relationship with. We attempted to account for this by only utilising PCVs who had been working in their community for 6 months or longer. The doctors in our study did not have the same difficulties as the older midwives in receiving the teaching. This could be explained as the doctors were generally younger, more educated, and aware of modern medicine and changing practice. Furthermore many had heard of Mercy Ships and so, knowing the teaching was being given in partnership with Mercy Ships may have given the program credibility. This may have helped the doctors recognise the training as valid even if it was being taught by younger, non-medical PCVs. Whereas, in contrast, most of the midwives had not heard of Mercy Ships and therefore perhaps did not recognise a higher degree of authority for the training other than simply the opinion of the young, non-medical PCV.

Our study has a number of limitations. It was a pilot study with a pragmatic choice of health centres based on geographical constraints. It was not randomised and neither was there a control group. Although we had a good follow up rate, 10/10 (100%) PCVs and 29/42 (70%) healthcare workers the numbers are small. The study is also open to bias because the PCVs who delivered the training also conducted the level 1 and 2 evaluations, though this is supported by similar methodology in other international courses run in low resource environments [20, 21]. Also, level 3 and 4 evaluations were carried out by Mercy Ships staff and since healthcare workers knew Mercy Ships had donated the bulb suction and self-inflating bags, the healthcare workers may have wanted to create a good impression by responding positively to the questions. A further limitation is the short-term nature of our follow up and lack of information on knowledge and skill retention, and change in practice beyond 4 months. Others have noted training may only have a short duration of impact and retraining at regular intervals is necessary [13]. However, the use of the PCV’s may mitigate some of the loss of knowledge/skill expected over time, as PCVs will continue to work in the rural health centres for a further 6–8 months post-training. Additionally since the HBB course was one of their focus projects, they may be able to reinforce training as needed with the HBB teaching materials supplied.

## Conclusion

We have shown that non-medically trained PCVs who have been in-country and working at a health centre for at least 6 months, can successfully be trained in the HBB method of newborn resuscitation and then they can in turn provide acceptable training to rural health care workers. This model of knowledge translation and implementation resulted in changes in practice of rural health care workers and qualitative, anecdotal evidence of improved patient outcome. This novel approach is important as it may be yet another tool in the armamentarium of those seeking to disseminate good medical practice to even the most rural areas.

## Additional file

Additional file 1: Anonymised raw data. (XLSX 16 kb)

## Abbreviations

HBB: Helping Babies breathe
PCV: Peace Corps volunteer
